# Implementation mapping to develop strategies for skin-to-skin care in the pediatric cardiac intensive care unit: planning for a hybrid trial

**DOI:** 10.3389/fped.2026.1851773

**Published:** 2026-06-09

**Authors:** Amanda Bettencourt, Jennifer Whittaker, Diya Nag, Katherine S. Kellom, Claire Brennan, Elizabeth A. Herrup, Abigail Demianczyk, Amy J. Lisanti

**Affiliations:** 1 Department of Family and Community Health, University of Pennsylvania School of Nursing, Philadelphia, PA, United States; 2 Qualitative Research Core, Research Institute, Children’s Hospital of Philadelphia, Philadelphia, PA, United States; 3 Cardiology Clinical Research, Division of Cardiology, Children’s Hospital of Philadelphia, Colket Translational Research Building, Philadelphia, PA, United States; 4 Division of Cardiac Critical Care Medicine, Department of Anesthesiology and Critical Care Medicine, Children’s Hospital of Philadelphia, University of Pennsylvania Perelman School of Medicine, Philadelphia, PA, United States; 5 Division of Pediatric Psychology, Department of Pediatric Behavioral Health & Neurosciences and Division of Cardiology & Cardiovascular Medicine, Department of Heart, Vascular & Thoracic Children’s Institute, Cleveland Clinic, Cleveland OH, United States; 6 Department of Family and Community Health, Research Institute, Children’s Hospital of Philadelphia, Philadelphia, PA, United States

**Keywords:** ERIC taxonomy, hybrid trial, implementation mapping, implementation science, kangaroo care, neonatal developmental care, PCICU, pediatric cardiac intensive care

## Abstract

**Background:**

Skin-to-skin care (SSC) is an evidence-based developmental practice that improves physiological stability, neurodevelopment, and parental well-being in hospitalized neonates. Although feasible and safe for neonates with complex congenital heart disease (cCHD) in the pediatric cardiac intensive care unit (PCICU), SSC remains underused due to knowledge gaps, unclear roles, safety concerns, inconsistent communication, and a lack of peer support.

**Methods:**

We used implementation mapping to identify determinants of SSC implementation in two United States (U.S.) PCICUs to design a targeted, theory-informed strategy package in preparation for a hybrid type II trial. Semi-structured interviews with clinicians (*n* = 25) and parents (*n* = 21) were analyzed using the Tailored Implementation for Chronic Diseases (TICD) framework. Determinants were linked to strategies using the Expert Recommendations for Implementing Change (ERIC) taxonomy. A logic model was developed to connect determinants, strategies, mechanisms, and outcomes.

**Results:**

Key barriers included limited SSC training, unclear responsibilities for initiation, safety concerns, inconsistent communication, and a lack of peer support. Three primary strategies were co-developed with stakeholders: (1) Education with role clarification, (2) Audit and feedback, and (3) Internal peer-to-peer facilitation. The logic model describes mechanisms such as improved knowledge, confidence, and role clarity; reinforcement through feedback; and peer modeling leading to improved adoption, feasibility, and sustainability of SSC.

**Conclusions:**

This streamlined, end-user informed strategy package addresses the main barriers to SSC implementation in the PCICU and offers a replicable model for linking determinants to strategies in acute care settings.

## Introduction

Skin-to-skin care (SSC)—placing a diapered infant directly on a caregiver's bare chest—has long been recognized as a fundamental practice of neonatal developmental care. Originally developed for preterm infants in resource-limited settings, SSC has since been widely implemented in neonatal intensive care units (NICUs) worldwide due to its proven benefits for infant physiological stability, growth, neurodevelopment, and parent-infant bonding (1–3). SSC also reduces neonatal physiological and behavioral responses to acute painful procedures (4, 5), a benefit particularly relevant in critical care environments where invasive interventions are routine. SSC also supports parental psychological well-being by reducing stress, anxiety, and symptoms of depression (6–8).

In neonates with complex congenital heart disease (cCHD), SSC offers similar benefits and has been shown to be safe and feasible in the pediatric cardiac intensive care unit (PCICU) (9–11). However, its adoption in PCICUs faces significant challenges, as documented in surveys of current practices (12–14). Previous research has shown that barriers are multifaceted (15) and include the higher acuity of patients, the presence of invasive lines and monitoring, the need for specialized clinical knowledge, and deeply ingrained workflows that prioritize medical care over developmental care. These obstacles are intensified by a lack of clear protocols, limited training opportunities, safety concerns, inconsistent communication between clinicians and parents, and a shortage of peer support for novice staff. These challenges echo well-documented barriers to SSC implementation across neonatal critical care contexts more broadly, where resource constraints, clinician knowledge and confidence gaps, safety concerns, and the absence of clear protocols have been consistently reported as pervasive determinants (16–19).

The underuse of SSC in the PCICU highlights a significant gap between evidence and practice. Implementation science offers tools to address these gaps by systematically identifying what influences practice and connecting these factors to strategies that overcome barriers and leverage facilitators (20–22). One such tool, implementation mapping, provides a step-by-step process for developing multi-level, theory-based implementation strategies tailored to specific contexts (20).

In a prior qualitative study, we examined the implementation determinants of SSC in two United States (U.S.) PCICUs through semi-structured interviews with clinicians and parents (15). Building on those findings, the aim of this study was to use implementation mapping to develop a targeted, theory-informed SSC implementation package for the PCICU setting to guide a future hybrid type II trial. The mapping process involved linking identified determinants to strategies using the Expert Recommendations for Implementing Change (ERIC) taxonomy (23), creating a logic model, and operationalizing strategies in preparation for testing.

## Materials and methods

### Design

Using data from a prior qualitative study examining SSC implementation determinants in the PCICU (15), we underwent an implementation mapping process, aligned with the five steps outlined by Fernandez et al. (20). The work was guided by the Tailored Implementation for Chronic Diseases (TICD) framework (24) to identify and organize determinants and by the Implementation Research Logic Model (22) to link determinants, strategies, mechanisms, and outcomes which followed the definitions from Proctor (21).

The five steps of implementation mapping articulated by Fernandez et al. (20) are: (1) conduct a needs assessment to identify adopters and implementers and characterize the implementation context; (2) state adoption and implementation outcomes, performance objectives, and determinants, and create matrices of change objectives; (3) choose theoretical methods (mechanisms of change) and select or design implementation strategies; (4) produce implementation protocols and materials; and (5) evaluate implementation outcomes. The present manuscript reports our application of steps 1 through 4; step 5 will be addressed in the planned hybrid type II trial. In step 1, we conducted a needs assessment using semi-structured interviews with PCICU clinicians and parents, analyzed with the TICD framework. In steps 2 and 3, we synthesized determinants, adoption and implementation outcomes, and theoretical mechanisms into an Implementation Research Logic Model (22), and engaged stakeholders to link determinants to evidence-based implementation strategies via the ERIC taxonomy (23). In step 4, we operationalized the selected strategies into a concrete, PCICU-specific protocol package.

The TICD framework (24) was selected as the organizing determinants framework for three reasons. First, it is a validated determinants framework that has demonstrated utility in implementation research conducted in acute and intensive care settings. Second, its seven categories—guideline factors, individual health professional factors, patient factors, professional interactions, incentives and resources, capacity for organizational change, and social, political, and legal factors—capture determinants across the individual, interpersonal, organizational, and system levels relevant to PCICU practice. Third, it was the determinants framework used in our prior qualitative study (15) from which this mapping work derives, enabling direct continuity between the empirically identified determinants and the strategies developed here.

### Setting and participants

The determinants study took place in two U.S. PCICUs:

**Site A:** A 38-bed PCICU in a quaternary academic children’s hospital that admits more than 115 neonates with cCHD undergoing neonatal cardiopulmonary bypass surgery annually, with an established SSC protocol and prior SSC implementation experience.
**Site B:** A 15-bed PCICU in an academic children’s hospital that admits approximately 45 neonates with cCHD undergoing neonatal cardiopulmonary bypass surgery annually, with minimal SSC implementation at study initiation.

Participants included:

**Clinicians:** Registered nurses, advanced practice providers (nurse practitioners and clinical nurse specialists), physicians, and respiratory therapists working in the PCICU.

**Parents:** Primary caregivers of neonates with cCHD hospitalized in the PCICU within the prior 6 months.

For clinicians, purposive sampling ensured diversity in discipline, years of experience, and prior SSC exposure. For parents, we purposively enrolled primary caregivers of neonates with cCHD hospitalized in the PCICU within the prior 6 months; electronic health records were screened for documentation of SSC during admission to prioritize recruitment of parents with direct SSC experience, and parents whose infants did not receive SSC during admission were also included to capture the full range of parental experience.

### Data collection

Semi-structured interview guides were developed for clinicians and parents, based on the TICD framework and previous SSC implementation research (12, 13). The interviews explored perceptions of SSC, perceived barriers and facilitators, communication styles, role clarity, and suggestions for increasing SSC adoption. Clinicians also shared their experiences with existing protocols and training.

Interviews were conducted via secure videoconferencing or telephone, audio-recorded with participants’ consent, and professionally transcribed. Demographic data were collected using brief questionnaires. Participant demographic characteristics are summarized in Supplementary Table S1, and the semi-structured interview guides used with clinicians and parents are provided in Supplementary File S2 [originally published with Lisanti et al. (15)].

## Data analysis

The qualitative analytic procedures used to identify the implementation determinants that anchor this mapping work are described in detail in our prior determinants publication (15). In brief, transcripts were analyzed using an integrated approach to qualitative content analysis (25) combining deductive coding based on the seven TICD framework domains with inductive coding of emergent themes; two team members independently coded each transcript subset, with formal inter-rater reliability assessment using Cohen's kappa. Average kappa was 0.94 for the clinician dataset and 0.95 for the parent dataset. The full study team met regularly to refine the codebook and resolve coding discrepancies by consensus.

Building on these determinants, implementation mapping Steps 2 through 4 were conducted by the research team with iterative feedback from key stakeholders. In Step 2, the team developed an Implementation Research Logic Model (22) that organized the TICD-derived determinants alongside hypothesized mechanisms, candidate strategies, and Proctor's implementation outcomes (21). In Step 3, the research team matched determinants to evidence-based strategies from the ERIC taxonomy (23), evaluating each candidate strategy for feasibility in the PCICU setting, expected impact across multiple outcomes, and alignment with established behavior change and organizational theories. Consistent with the Pareto principle (26), the team prioritized the “vital few” strategies anticipated to drive change across the most outcomes. The draft logic model and selected strategies were then shared with key stakeholders from multiple groups—including multi-disciplinary PCICU clinicians and parents with prior experience of having an infant cared for in the PCICU—who reviewed the materials and confirmed that the chosen strategies appropriately addressed the identified determinants. In Step 4, the validated strategies were operationalized into a concrete, PCICU-specific protocol package, with each strategy linked to its underlying theoretical mechanism and translated into bedside-actionable elements (educational content, audit and feedback artifacts, champion role specifications).

## Ethics

The study was reviewed and granted exempt status by the Institutional Review Boards of both participating institutions (Children's Hospital of Philadelphia, IRB #23-021241; Cleveland Clinic, IRB #24-258). Written or verbal informed consent was obtained from all participants.

## Results

### Step 1: needs assessment

Interviews with 25 clinicians and 21 parents across two PCICUs identified SSC implementation determinants at various TICD framework levels.

**Clinician perspectives** indicated that while SSC was viewed positively and aligned with family-centered care principles, there were notable gaps in knowledge of formal SSC guidelines, patient eligibility criteria, and safe methods for performing SSC with neonates requiring complex monitoring or support devices. Safety concerns were prominent, particularly regarding the risk of accidental device dislodgement or physiologic instability. Many clinicians expressed uncertainty about whether they had the authority to initiate SSC or if physician approval was needed.

**Parent perspectives** emphasized a strong motivation to provide SSC, often describing it as an important bonding experience. However, parents frequently received inconsistent messages from different team members about when SSC was permitted, leading to confusion and missed opportunities. Several parents expressed hesitation to ask about SSC, fearing they would impose additional demands on staff.

Across both sites, clinician participants noted a lack of peer support for less experienced staff as a barrier to consistently initiating SSC. This was especially true for staff who had not personally observed or assisted with SSC in the PCICU setting.

**Figure 1:**
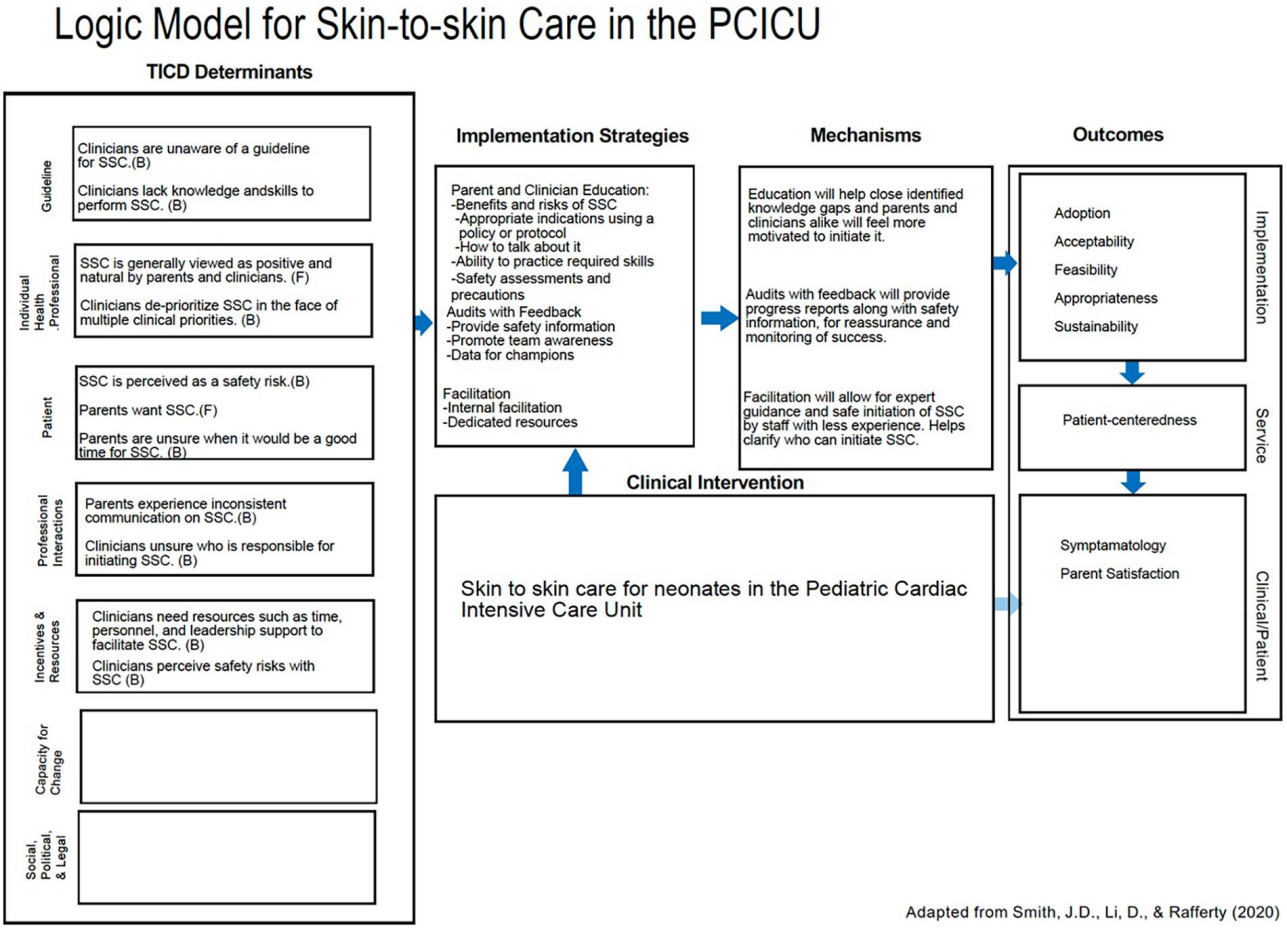
Implementation research logic model for skin-to-skin care in the pediatric cardiac intensive care unit (PCICU). The model connects identified determinants of SSC implementation to selected strategies, hypothesized mechanisms, and anticipated implementation and patient outcomes. In the determinants column, **(B)** indicates a barrier and **(F)** indicates a facilitator.

Across both stakeholder groups, six key implementation determinants were identified that informed all subsequent mapping steps (Steps 2–4): limited awareness of SSC protocols and guidelines; insufficient SSC-specific training for managing high-acuity patients; safety concerns related to patient stability and device security; unclear role and responsibility for SSC initiation; inconsistent communication between clinicians and parents; and a lack of peer modeling and support for novice staff. These determinants spanned five TICD framework domains (guideline factors, individual health professional factors, patient factors, professional interactions, and incentives and resources), as displayed in Figure 1. Facilitators included positive attitudes toward SSC among parents and clinicians and strong parental desire to participate in SSC when given the opportunity. The full set of determinants and supporting findings is reported in detail in our prior determinants publication (15).

### Step 2: develop logic model

Findings from Step 1 were then organized into an Implementation Research Logic Model tailored for SSC in the PCICU. The logic model was developed iteratively by the research team; consultation with and validation by key stakeholders occurred during Step 3 (described below). The final logic model connected identified determinants to the implementation strategies subsequently selected in Step 3, hypothesized mechanisms of action, and anticipated implementation and patient outcomes (Figure 1).

### Step 3: operationalize implementation strategies

#### Strategy selection and stakeholder validation

The research team mapped each identified determinant to candidate strategies from the ERIC taxonomy (23), examining each candidate strategy for its underlying mechanism of action, feasibility within the PCICU setting, and pathways to operationalization. The resulting logic model and strategy package were then shared with key stakeholders from multiple groups—including multi-disciplinary PCICU clinicians and parents with prior experience of an infant cared for in the PCICU—who reviewed the materials and validated that the chosen strategies appropriately addressed the identified determinants. Stakeholders confirmed the appropriateness of the package prior to operationalization in Step 4.

#### Final strategies selected

While many discrete implementation strategies emerged, three primary strategies were chosen for their high feasibility, solid theoretical foundation, and anticipated impact across multiple outcomes. The research team applied the three explicit criteria above (mechanism of action, feasibility in the PCICU setting, and pathways to operationalization) through iterative discussion to narrow the initial candidate list to the three strategies most likely to drive change across the largest number of determinants and outcomes. No formal voting or structured consensus methodology (such as Delphi or nominal group technique) was used; the final selection was reached by research team consensus and subsequently validated by stakeholders as described above. This is consistent with the Pareto principle (26) of choosing the “vital few” that will generate change in the most outcomes.

**1. Education with role clarification.** Purpose: Address gaps in knowledge and skills, and clarify responsibilities for SSC initiation. Operationalization: Conduct in-person and online SSC training for all staff; include a protocol explicitly naming the RN as the SSC initiator; use laminated bedside reference sheets; hold annual simulation exercises with mannequins; provide parent-facing brochures and videos describing the SSC process, benefits, and safety measures.
**2. Audit and feedback.** Purpose: Monitor SSC frequency and safety to reassure staff and promote participation. Operationalization: Monthly SSC initiation and safety reports shared with staff and leadership; visual dashboards in staff areas display current performance and trends.
**3. Internal peer-to-peer facilitation.** Purpose: Deliver bedside coaching and real-time troubleshooting through trained SSC champions. Operationalization: Assign a SSC champion (among existing staff) on each shift to support initial SSC sessions, demonstrate safe techniques, assist with device management, and reinforce protocols.

#### Link to theory

Strategies were explicitly connected to relevant theories: Social Cognitive Theory (27) (skill acquisition through modeling and reinforcement), Diffusion of Innovations (28) (leveraging champions to promote adoption), and Organizational Development Theory (29) (integrating SSC into team culture and workflows).

### Step 4: protocolize implementation strategies

Interview findings highlighted that the strategy package should be straightforward, actionable, and resource-efficient for integration into busy PCICU workflows. The final package is summarized in Table 1. The mapping from determinants to strategies is presented in Table 2, and representative participant quotes are provided in Table 3. The present manuscript reports the strategy-level operationalization developed through implementation mapping. Full protocol-level specification of each strategy—including specific delivery personnel, dose, frequency, recipient touchpoints, fidelity measures, and additional site-specific contextual tailoring—will be reported in the forthcoming hybrid type II effectiveness-implementation trial protocol publication, which corresponds to Step 5 of the implementation mapping process.

**Table 1:** Final SSC implementation strategy menu.

Strategy category	Implementation strategy	Operationalization example
Educate	Education with role clarification	SSC training during staff meetings and orientation; protocol assigning SSC initiation to RNs; laminated bedside checklist; parent education materials
Evaluate	Audit and feedback	Monthly SSC initiation and safety data shared with staff and leadership
Assist	Internal peer-to-peer facilitation	SSC champions provide bedside coaching for initial SSC sessions and support during shift huddles

This strategy menu represents the final three-component implementation package developed through stakeholder collaboration and theoretical mapping.

SSC, skin-to-skin care; RN, registered nurse.

**Table 2:** Determinants, matched strategies, operational definitions, and theoretical rationale.

Determinant (TICD domain)	Strategy	Strategy definition	Operationalization for SSC in the PCICU	Relevant theory
Clinicians unaware of SSC guideline (Individual/Guideline)	Education with role clarification	Provide targeted training and clarify responsibilities for SSC initiation	In-person and online SSC training modules for all PCICU staff; protocol specifying RN as primary SSC initiator with team support	Social Cognitive Theory; Organizational Development Theory
Clinicians lack SSC skills (Individual)	Education with role clarification	Provide competency-based practice and clear initiation roles	Annual SSC simulation; bedside job aids; visual role charts	Social Cognitive Theory
SSC perceived as safety risk (Individual/Process)	Audit and feedback	Share performance data to reinforce safety and uptake	Monthly SSC incidence and safety reports	Social Cognitive Theory
Parents unsure when SSC is appropriate (Patient/Professional Interaction)	Education with role clarification	Provide accessible SSC information to families; clarify team roles	Parent brochures/videos; nurse-led discussions on eligibility	Health Belief Model
Inconsistent SSC communication (Professional Interaction)	Internal peer-to-peer facilitation	Use trained SSC champions to coordinate and coach peers	Champions lead bedside support for initial SSC sessions and promote discussion during shift huddles	Diffusion of Innovations
Unclear role responsibility for SSC initiation (Professional Interaction)	Education with role clarification	Define and communicate initiation responsibilities	Protocol embedded into orientation and annual competency training	Organizational Development Theory
Lack of clinician confidence to manage devices during SSC (Individual)	Internal peer-to-peer facilitation	Provide real-time bedside guidance from experienced peers	Champions coach clinicians through device management during SSC	Social Cognitive Theory

SSC, skin-to-skin care; PCICU, pediatric cardiac intensive care unit; RN, registered nurse; TICD, Tailored Implementation for Chronic Diseases framework.

**Table 3:** Representative quotes by determinant and strategy.

Determinant	Representative quote (shortened)	Linked strategy
Clinicians unaware of SSC guideline	"I didn't even know we had a skin-to-skin protocol until last year."	Education with role clarification
Clinicians lack SSC skills	"Some nurses haven't been educated… don't know patients can be held with devices."	Education with role clarification
SSC perceived as safety risk	"Want to know which patients had complications to avoid them."	Audit and feedback
Parents unsure when SSC is appropriate	"I wasn't sure if I could do skin-to-skin — no one told me."	Education with role clarification
Inconsistent SSC communication	"Unless the family brings it up… it doesn't get talked about."	Internal peer-to-peer facilitation
Unclear role responsibility for SSC initiation	"I trust my nurses to guide those topics."	Education with role clarification
Lack of clinician confidence to manage devices	"Only the lactation consultant encouraged skin-to-skin."	Internal peer-to-peer facilitation

Quotes are from semi-structured interviews with clinicians and parents across two pediatric cardiac intensive care units (PCICUs). Quotes have been shortened for brevity while preserving meaning and context.

SSC, skin-to-skin care.

## Discussion

This study employed a structured implementation mapping process (20) to develop a targeted, context-specific strategy package aimed at increasing the use of skin-to-skin care (SSC) for neonates with complex congenital heart disease (cCHD) in the pediatric cardiac intensive care unit (PCICU). Through end-user engagement, determinants were identified, linked to strategies, and organized into a logic model that connects obstacles and facilitators with hypothesized mechanisms and outcomes. The resulting three-primary-strategy package—Education with role clarification, Audit and feedback, and Internal peer-to-peer facilitation—is based on implementation theory and specifically tailored to the challenges of the PCICU setting.

## Comparison with prior SSC implementation research

In NICUs, SSC implementation strategies have often focused solely on education (1–3), with mixed results. While clinician training increases awareness and knowledge, evidence from both neonatal and pediatric settings shows that education alone does not suffice to change practice (30–32). The current study expands on this by combining education with clear role clarification, ensuring that the responsibility for initiating SSC is explicitly assigned to a specific team member (the RN in our model). This aligns with findings from other developmental care efforts in critical care, where clear delegation of responsibilities enhances adoption and sustainability (33).

Audit and feedback have been less frequently used in SSC but have demonstrated effectiveness in other critical care interventions (34–36). Our findings suggest that providing regular, transparent data on SSC initiation and safety not only educates staff but also alleviates safety concerns—a barrier consistently reported in both NICU and PCICU settings (12, 13). Recent reviews further suggest that audit and feedback interventions are most effective when explicitly grounded in behavior change theory (37), an alignment we incorporated by linking this strategy to Social Cognitive Theory.

Internal peer-to-peer facilitation—utilizing peer facilitators to coach and demonstrate best practices—has been widely acknowledged as a key factor in successful implementation across different healthcare settings (38–40). In the PCICU environment, where high acuity and complexity may discourage novice staff from starting SSC, facilitators help normalize the practice, troubleshoot in real time, and incorporate SSC into daily workflows. This aligns with Social Cognitive Theory, where observation and modeling are fundamental mechanisms of behavior change (27).

## Theoretical rationale for the three-strategy package

The strategy package deliberately integrates several components, drawing from different theoretical perspectives to target factors at the individual, interpersonal, and organizational levels.

Social Cognitive Theory (27) underpins both education and facilitation aspects, emphasizing the importance of skill development, self-efficacy, and observational learning. Diffusion of Innovations Theory (28) guides the champion model, recognizing that visible early adopters can accelerate adoption. Organizational Development Theory (29) helps clarify roles and integrate SSC into standard workflows, supporting sustainability. The Health Belief Model (41) informs family education by addressing perceptions of benefits and barriers to SSC.

This theoretical triangulation aligns with implementation science recommendations for targeting multiple mechanisms simultaneously to maximize adoption and sustainment (42, 43). Our application of Pareto principles (26) in choosing three primary strategies aligns with current recommendations to avoid so-called “Cadillac” or multiple multilevel strategy-packaged implementation interventions and to focus on the most pragmatic solutions to improve care outcomes (44).

## Implications for practice

The SSC strategy package developed here includes several features that enhance its practical application.

First, the strategies demonstrate feasibility in high-acuity settings because they are designed to integrate into existing workflows, which reduces additional burden on staff. Second, responsibilities are clearly defined by explicitly assigning initiation to RNs, minimizing confusion and promoting accountability. Third, ongoing reinforcement through audits, feedback, and peer facilitation offers continuous opportunities for improvement and reassurance. Fourth, family engagement is strengthened through educational materials for parents that support a family-centered care approach, encouraging shared decision-making.

These features are not exclusive to SSC and could be adapted for other developmental care interventions in high-acuity pediatric settings.

## Implications for research

The next step will be to evaluate the package's impact on outcomes within a hybrid trial that will assess both clinical effectiveness (e.g., SSC frequency, infant physiological outcomes, parent mental health) and implementation outcomes (adoption, acceptability, feasibility, appropriateness, sustainability) (45). It will also explore whether the proposed mechanisms—improved knowledge, role clarity, safety confidence, and peer support—serve as mediators in the relationship between strategies and SSC adoption.

Additionally, the current study contributes to the growing literature on implementation mapping (20) in acute care settings, demonstrating that the process can be effectively adapted to environments with high patient acuity and complex interprofessional teams.

## Strengths and limitations

Strengths of this study include using a validated determinant framework (TICD) to guide data collection and analysis, engaging both parent and clinician stakeholders from two different PCICU settings, clearly connecting determinants to strategies through the ERIC taxonomy, and operationalizing each strategy based on theory.

Limitations include that findings only apply to two PCICU settings and might not extend to all institutions. Additionally, determinants were identified through self-report, which might be influenced by recall and social desirability biases. Although the strategies were deemed feasible by the research and stakeholder team, the actual implementation was not assessed in this phase.

## Conclusion

Using a systematic implementation mapping process (20), we developed a targeted, theory-informed, pragmatic strategy package to promote skin-to-skin care (SSC) for neonates with complex congenital heart disease (cCHD) in the pediatric cardiac intensive care unit (PCICU). The package—comprising education with role clarification, audit and feedback, and internal peer-to-peer facilitation—addresses the major barriers to SSC adoption identified by clinicians and parents across two diverse PCICU settings. Based on implementation science frameworks and theories, the package is designed for feasibility, sustainability, and flexibility within high-acuity care environments.

This work not only offers a practical, stakeholder-supported roadmap for enhancing SSC implementation in the PCICU but also serves as another example of the use of implementation mapping in acute care settings. The next step is to evaluate this package in planning a hybrid trial, assessing both implementation and clinical outcomes.

## Data Availability

The datasets generated and/or analyzed during the current study are not publicly available due to participant confidentiality but can be provided by the corresponding author upon reasonable request and with proper institutional approval.
