# Supplementary material for: Implementation mapping to develop strategies for skin-to-skin care in the pediatric cardiac intensive care unit: planning for a hybrid trial
Source: Front Pediatr. 2026 Jun 9;14:1851773. doi: 10.3389/fped.2026.1851773 (PMC13286826; doi:10.3389/fped.2026.1851773)
Supplement: Supplementary file 1 [file Table1.docx]

# Supplementary Table S1. Participant Characteristics

*Demographic and professional characteristics of parent (n = 21) and clinician (n = 25) participants in the implementation determinants study underlying this implementation mapping work. Adapted from Lisanti et al., Journal of Pediatric Nursing 2025;85:321–331.*

**Panel A. Parent participants (n = 21)**

| **Characteristic** | **Site A (n = 15)** | **Site B (n = 6)** | **Total (n = 21)** |
| --- | --- | --- | --- |
| ***Continuous variables, mean (SD)*** | | | |
| Parent age (years) | 33.5 (5.4) | 30.5 (8.1) | 32.7 (6.3) |
| Timing of cCHD diagnosis (weeks gestation)¹ | 20.4 (4.7) | 24.0 (8.2) | 21.3 (5.8) |
| Infant hospital length of stay (days) | 63.8 (81.6) | 36.1 (20.1) | 55.9 (70.2) |
| ***Categorical variables, n (%)*** | | | |
| *Gender* |  |  |  |
| Female | 14 (93.3) | 6 (100) | 20 (95.2) |
| Male | 1 (6.7) | 0 (0) | 1 (4.8) |
| *Marital status* |  |  |  |
| Single | 2 (13.3) | 2 (33.3) | 4 (19.0) |
| Married | 12 (80.0) | 4 (66.7) | 16 (76.2) |
| Domestic partner | 1 (6.7) | 0 (0) | 1 (4.8) |
| *Race* |  |  |  |
| White | 11 (73.3) | 3 (50.0) | 14 (66.7) |
| Black | 2 (13.3) | 1 (16.7) | 3 (14.3) |
| Multi | 0 (0) | 1 (16.7) | 1 (4.8) |
| Asian / Pacific Islander / American Indian / Alaska Native | 2 (13.3) | 1 (16.7) | 3 (14.3) |
| *Ethnicity* |  |  |  |
| Hispanic | 1 (6.7) | 0 (0) | 1 (4.8) |
| Non-Hispanic | 14 (93.3) | 6 (100) | 20 (95.2) |
| *Main health insurance* |  |  |  |
| Public insurance | 3 (20.0) | 4 (66.7) | 7 (33.3) |
| Private insurance | 12 (80.0) | 2 (33.3) | 14 (66.7) |
| *Highest level of education completed* |  |  |  |
| Partial high school | 0 (0) | 1 (16.7) | 1 (4.8) |
| Graduated from high school | 2 (13.3) | 1 (16.7) | 3 (14.3) |
| Technical / vocational school | 2 (13.3) | 0 (0) | 2 (9.5) |
| Partial college | 2 (13.3) | 2 (33.3) | 4 (19.0) |
| College graduate | 8 (53.3) | 1 (16.7) | 9 (42.9) |
| Doctoral degree | 1 (6.7) | 1 (16.7) | 2 (9.5) |
| *Timing of cCHD diagnosis* |  |  |  |
| Prenatal | 14 (93.3) | 5 (83.3) | 19 (90.5) |
| Postnatal | 1 (6.7) | 1 (16.7) | 2 (9.5) |
| *First birth* |  |  |  |
| No | 8 (53.3) | 5 (83.3) | 13 (61.9) |
| Yes | 7 (46.7) | 1 (16.7) | 8 (38.1) |
| *SSC experience during hospitalization* |  |  |  |
| No | 4 (26.7) | 0 (0) | 4 (19.0) |
| Yes | 11 (73.3) | 6 (100) | 17 (81.0) |
| *Infant sex* |  |  |  |
| Female | 6 (40.0) | 1 (16.7) | 7 (33.3) |
| Male | 9 (60.0) | 5 (83.3) | 14 (66.7) |

*¹ Two parents did not report the cCHD diagnosis week; no other missingness observed.*

**Panel B. Clinician participants (n = 25)**

| **Characteristic** | **Site A (n = 15)** | **Site B (n = 10)** | **Total (n = 25)** |
| --- | --- | --- | --- |
| ***Continuous variables, mean (SD)*** | | | |
| Years of experience in current role | 5.6 (4.7) | 10.8 (7.1) | 7.7 (6.2) |
| Years of experience in profession | 12.7 (10.4) | 13.4 (6.2) | 13.0 (8.8) |
| Age (years) | 37.8 (10.1) | 41.7 (8.6) | 39.4 (9.5) |
| ***Categorical variables, n (%)*** | | | |
| *Working hours per week* |  |  |  |
| At least 36 h per week | 12 (80.0) | 9 (90.0) | 21 (84.0) |
| 20 to 35 h per week | 3 (20.0) | 1 (10.0) | 4 (16.0) |
| *Role* |  |  |  |
| Bedside nurse | 5 (33.3) | 2 (20.0) | 7 (28.0) |
| Nurse manager or administrator | 1 (6.7) | 1 (10.0) | 2 (8.0) |
| Clinical nurse specialist | 1 (6.7) | 0 (0) | 1 (4.0) |
| Nurse educator | 1 (6.7) | 1 (10.0) | 2 (8.0) |
| Nurse practitioner | 1 (6.7) | 2 (20.0) | 3 (12.0) |
| Fellow / resident | 1 (6.7) | 0 (0) | 1 (4.0) |
| Critical care attending | 2 (13.3) | 1 (10.0) | 3 (12.0) |
| Cardiologist attending | 0 (0) | 1 (10.0) | 1 (4.0) |
| Cardiothoracic surgery attending | 2 (13.3) | 2 (20.0) | 4 (16.0) |
| Respiratory therapist | 1 (6.7) | 0 (0) | 1 (4.0) |
| *Gender* |  |  |  |
| Female | 12 (80.0) | 7 (70.0) | 19 (76.0) |
| Male | 3 (20.0) | 3 (30.0) | 6 (24.0) |
| *Race* |  |  |  |
| Asian | 1 (6.7) | 0 (0) | 1 (4.0) |
| Black / African American | 1 (6.7) | 1 (10.0) | 2 (8.0) |
| Pacific Islander | 1 (6.7) | 0 (0) | 1 (4.0) |
| White | 11 (73.3) | 9 (90.0) | 20 (80.0) |
| Other | 1 (6.7) | 0 (0) | 1 (4.0) |
| *Ethnicity* |  |  |  |
| Hispanic | 2 (13.3) | 0 (0) | 2 (8.0) |
| Non-Hispanic | 12 (80.0) | 10 (100) | 22 (88.0) |
| Other | 1 (6.7) | 0 (0) | 1 (4.0) |
| *Highest level of completed education* |  |  |  |
| Bachelor’s degree | 4 (26.7) | 3 (30.0) | 7 (28.0) |
| Master’s degree | 5 (33.3) | 2 (20.0) | 7 (28.0) |
| Doctoral degree | 6 (40.0) | 5 (50.0) | 11 (44.0) |
| *SSC order requirement* |  |  |  |
| Yes – order required | 2 (13.3) | 1 (10.0) | 3 (12.0) |
| No – no order required | 7 (46.7) | 4 (40.0) | 11 (44.0) |
| I don’t know | 6 (40.0) | 4 (40.0) | 10 (40.0) |
| Other² | 0 (0) | 1 (10.0) | 1 (4.0) |
| *SSC support in past month³* |  |  |  |
| None | 8 (57.1) | 2 (20.0) | 10 (41.7) |
| 1–3 times | 6 (42.9) | 6 (60.0) | 12 (50.0) |
| 6–10 times | 0 (0) | 2 (20.0) | 2 (8.3) |

*² Participant response: “We currently don’t do SSC.”*

*³ One missed report for the number of SSC support events in the past month.*

**Abbreviations:** cCHD, complex congenital heart disease; PCICU, pediatric cardiac intensive care unit; SD, standard deviation; SSC, skin-to-skin care.
