# Supplementary material for: Implementation mapping to develop strategies for skin-to-skin care in the pediatric cardiac intensive care unit: planning for a hybrid trial
Source: Front Pediatr. 2026 Jun 9;14:1851773. doi: 10.3389/fped.2026.1851773 (PMC13286826; doi:10.3389/fped.2026.1851773)
Supplement: Supplementary file 2 [file Table2.docx]

# Supplementary File S2. Semi-Structured Interview Guides

*The semi-structured interview guides used with clinicians and parents in the implementation determinants study underlying this implementation mapping work are reproduced below. These guides were originally published as supplemental material with Lisanti et al., Journal of Pediatric Nursing 2025;85:321–331.*

## **Parent Interview Guide**

**Intro:** Skin-to-skin care, sometimes referred to as Kangaroo Care, is a form of holding where parents hold their unclothed, diapered baby directly to their chest. For this interview, we want to understand more about your personal perceptions regarding the use of skin-to-skin care in the PCICU, thinking about your needs and how the care team might play a role in supporting or not supporting skin-to-skin care. We are separate from your care team and none of the information you share will be shared directly with your care team and will remain anonymous. We value your feedback and we understand that any feedback you provide will help us to do a better job, but doesn’t mean you were not happy with the care you received.

**1.** To start off, how do you as a parent view SSC in general? Or what were your thoughts about SSC going into delivery / how was SSC a part of your birth plan? What were you told would be possible? Did that aspect of the birthing plan happen as you wanted?

**2.** When you were in the PCICU, where was skin-to-skin care on your priority list? Did your preferences or plan change once your baby was in the PCICU? Did you hold your baby? When and how often? Did you hold skin-to-skin while in the PCICU?

- How did that feel? (to practice skin-to-skin / to not practice skin-to-skin)
- How did participating or not participating in SSC affect you and your baby?

**3.** Tell me about the things that influenced your ability to hold or not hold your baby skin-to-skin:

- Your child’s needs? (Did you notice your baby behaving in ways that discouraged you or PCICU clinicians from supporting SSC?)
- Your preferences?
- Your PCICU clinicians? (Did you feel supported or discouraged by PCICU clinicians to hold your baby skin-to-skin?)
- PCICU culture or norms? (Did you perceive that PCICU clinicians have beliefs/values that are different from your own regarding SSC, e.g., prevailing norms, opinions of colleagues, accepted behaviors?)
- PCICU policies on SSC?
- Other parents’ experiences in the PCICU?

**4.** How supported did you feel in having the SSC experience you wanted?

- What contributed to that feeling of support?

**5.** What didn’t feel supportive?

**6.** To support individual or family preferences around SSC, I’d love to hear about any feedback you have for things that might be done differently to support families like yours:

- What knowledge or education could be given to parents to support their preferences for SSC when your baby is in the PCICU?
- Do you feel clinicians had the resources needed to support you and the care team making a decision together regarding skin-to-skin? If yes, what was helpful? If not, what is needed? (e.g., human resources / staffing, facilities, equipment and supplies, decision aids / support, guidelines / procedures, or clinical supervision)

## **Clinician Interview Guide**

**Intro:** We are going to be talking about skin-to-skin care during this interview. For the purpose of our discussion, skin-to-skin care, sometimes referred to as Kangaroo Care, is a form of holding where parents hold their unclothed, diapered baby directly to their bare chest. For this interview, we want to understand more about your perceptions regarding the use of skin-to-skin care in the PCICU for infants with congenital heart disease and their parents.

**1.** To start this conversation, tell me the protocols or guidelines that are in place in your PCICU, if any.

**2.** How do you view SSC as an intervention in the PCICU? Is there an experience you have had with SSC that influences your attitude or belief about SSC?

**3.** How do clinicians working in your PCICU view SSC in general? Is it different by role or experience? Is there a unit culture influencing these perspectives? Do you feel anything should be done differently — when, where, how, how often, and with whom?

**4.** We understand rules and protocols only go so far. Can you talk more about the team dynamics and communication between PCICU clinicians that influence SSC practice?

**5.** Can you give a case example that you remember?

**6.** What are some of the factors that influence your decision to have a parent provide SSC or not in the PCICU?

- What things relate to the patient?
- What things relate to the workflow?
- What things relate to the parents?
- What things relate to cultural norms of the unit?

**7.** What resources are needed to support the care team making a decision together with parents regarding skin-to-skin during the PCICU admission? What resources do you need to support those decisions? (e.g., human resources / staffing, facilities, equipment and supplies, decision aids / support, guidelines / procedures, or clinical supervision)

**8.** If PCICU clinicians and parents have different values regarding SSC, how is that resolved, if at all?

**9.** As we are thinking about supporting parent preferences and the needs of these infants, what knowledge, expertise, skills, or resources do PCICU clinicians need? Is it different by role or experience?
